# Salivary Cytoprotective Proteins in Inflammation and Resolution during Experimental Gingivitis—A Pilot Study

**DOI:** 10.3389/fcimb.2015.00092

**Published:** 2016-01-05

**Authors:** Guy M. Aboodi, Corneliu Sima, Eduardo B. Moffa, Karla T. B. Crosara, Yizhi Xiao, Walter L. Siqueira, Michael Glogauer

**Affiliations:** ^1^Department of Periodontology and Matrix Dynamics Group, Faculty of Dentistry, University of TorontoToronto, ON, Canada; ^2^Department of Biochemistry and Schulich Dentistry, Schulich School of Medicine and Dentistry, The University of Western OntarioLondon, ON, Canada; ^3^Department of Prosthodontics, CEUMA UniversitySao Luis, Brazil

**Keywords:** gingivitis, proteomics, saliva, oral neutrophils, mass spectrometry, inflammation

## Abstract

**Objective:** The protective mechanisms that maintain periodontal homeostasis in gingivitis and prevent periodontal tissue destruction are poorly understood. The aim of this study was to identify changes in the salivary proteome during experimental gingivitis.

**Study design:** We used oral neutrophil quantification and whole saliva (WS) proteomics to assess changes that occur in the inflammatory and resolution phases of gingivitis in healthy individuals. Oral neutrophils and WS samples were collected and clinical parameters measured on days 0, 7, 14, 21, 28, and 35.

**Results:** Increased oral neutrophil recruitment and salivary cytoprotective proteins increased progressively during inflammation and decreased in resolution. Oral neutrophil numbers in gingival inflammation and resolution correlated moderately with salivary β-globin, thioredoxin, and albumin and strongly with collagen alpha-1 and G-protein coupled receptor 98.

**Conclusions:** Our results indicate that changes in salivary cytoprotective proteins in gingivitis are associated with a similar trend in oral neutrophil recruitment and clinical parameters.

**Clinical relevance:** We found moderate to strong correlations between oral neutrophil numbers and levels of several salivary cytoprotective proteins both in the development of the inflammation and in the resolution of gingivitis. Our proteomics approach identified and relatively quantified specific cytoprotective proteins in this pilot study of experimental gingivitis; however, future and more comprehensive studies are needed to clearly identify and validate those protein biomarkers when gingivitis is active.

## Introduction

Periodontal diseases are a diverse group of inherited or acquired conditions that affect the tooth-supporting tissues in more than half of world population. Different pathogenic mechanisms including inflammatory, traumatic, genetic, and neoplastic contribute to the onset and progression of periodontal diseases (Madianos et al., 2005). The main etiologic factor for these conditions is the bacterial biofilm while the most common forms of periodontal diseases are plaque-induced gingivitis (GI) and chronic periodontitis (CP).

Plaque-induced gingivitis (GI) is defined as an inflammation of the gingiva induced by bacteria located at the gingival margin. The causative relationship between bacterial plaque (biofilm) and gingival inflammation was well demonstrated in experimental gingivitis (Loe et al., 1965). Characteristic GI clinical signs include erythema, edema, loss of gingival stippling, and bleeding upon probing (Mariotti, 1999). Interestingly, the host response to similar plaque levels varies significantly among patients (Trombelli et al., 2004). Histologic changes in the tissue include proliferation of junctional epithelium, vasculitis of blood vessels adjacent to the junctional epithelium, collagen degradation, cytopathologic alteration of fibroblasts, and inflammatory infiltrate (Page and Schroeder, 1976). GI is reversible upon removal of the etiologic biofilm (Loe et al., 1965). CP is characterized by extension of gingival inflammation to the alveolar bone, connective tissue degradation, and net loss of tooth attachment to periodontium (American Academy of Periodontology, 2000). The transition from GI to CP is incompletely understood. On the one hand, GI is an established risk factor for CP (Lang et al., 2009). On the other hand, clinical studies have demonstrated that in some individuals GI never progresses to CP, regardless of periodontal care (Pihlstrom et al., 2005).

Although certain pathogenic bacteria in subgingival biofilms produce specific virulence factors that could cause direct damage to periodontal tissues, current evidence suggest that it is the host factors that drive periodontal tissue degradation at sites with CP. These factors include an increase abundance of inflammatory cytokines, host proteolytic enzymes, and increased oxidative stress (Chapple and Matthews, 2007; Bartold et al., 2010). The rate limiting steps in onset and progression of clinical attachment loss are incompletely understood. Increasing evidence that emerged in recent years indicates that failure to resolve biofilm-induced periodontal inflammation results in chronicity and pro-osteolytic environments (Bartold and Van Dyke, 2013; Freire and Van Dyke, 2013; Van Dyke, 2014). Well-functioning resolution programs in periodontal tissues may be more critical that in other tissues because as a result of continuous challenge by subgingival bacteria, neutrophils traffic between the vasculature, gingival tissues, and gingival crevicular fluid (GCF) to maintain the host–biofilm balance, prevent tissue invasion by pathogens and ultimately bone loss.

The development of proteomics techniques allows us to identify, characterize and quantitate large numbers of proteins in a single study (Neilson et al., 2011). Utilizing proteomics in periodontitis research may reveal changes in the protein profile (proteome) during disease progression and the identification of disease-specific biomarkers (Baliban et al., 2012). WS and GCF samples were used for proteome analysis during GI (Ozdemir et al., 2009; Grant et al., 2010; Gonçalves Lda et al., 2011) and CP (Gonçalves Lda et al., 2010; Baliban et al., 2012; Rangé et al., 2012; Salazar et al., 2013; Silva-Boghossian et al., 2013). Blood proteins levels were reported to increase in both CP and GI patients (Gonçalves Lda et al., 2010, 2011), confirming the inflammatory nature of both diseases. While GCF and tissue samples provide site-specific information, salivary proteome analysis provides a comprehensive approach to oral health status, as WS proteins are originated from numerous sources (salivary glands, mucosal cells, immune cells, serum, and bacteria). The purpose of the current investigation was to identify changes in proteome profile during the development, progression and resolution of GI in an EG (experimental gingivitis) model. Findings of specific cytoprotective proteins and their correlation with clinical parameters and oral neutrophil numbers may suggest different protective mechanisms during GI that prevent tissue destruction and attachment loss that can be used for disease activity biomarker characterization and new preventive therapies for CP.

## Materials and methods

### Study design

**Figure 1:**
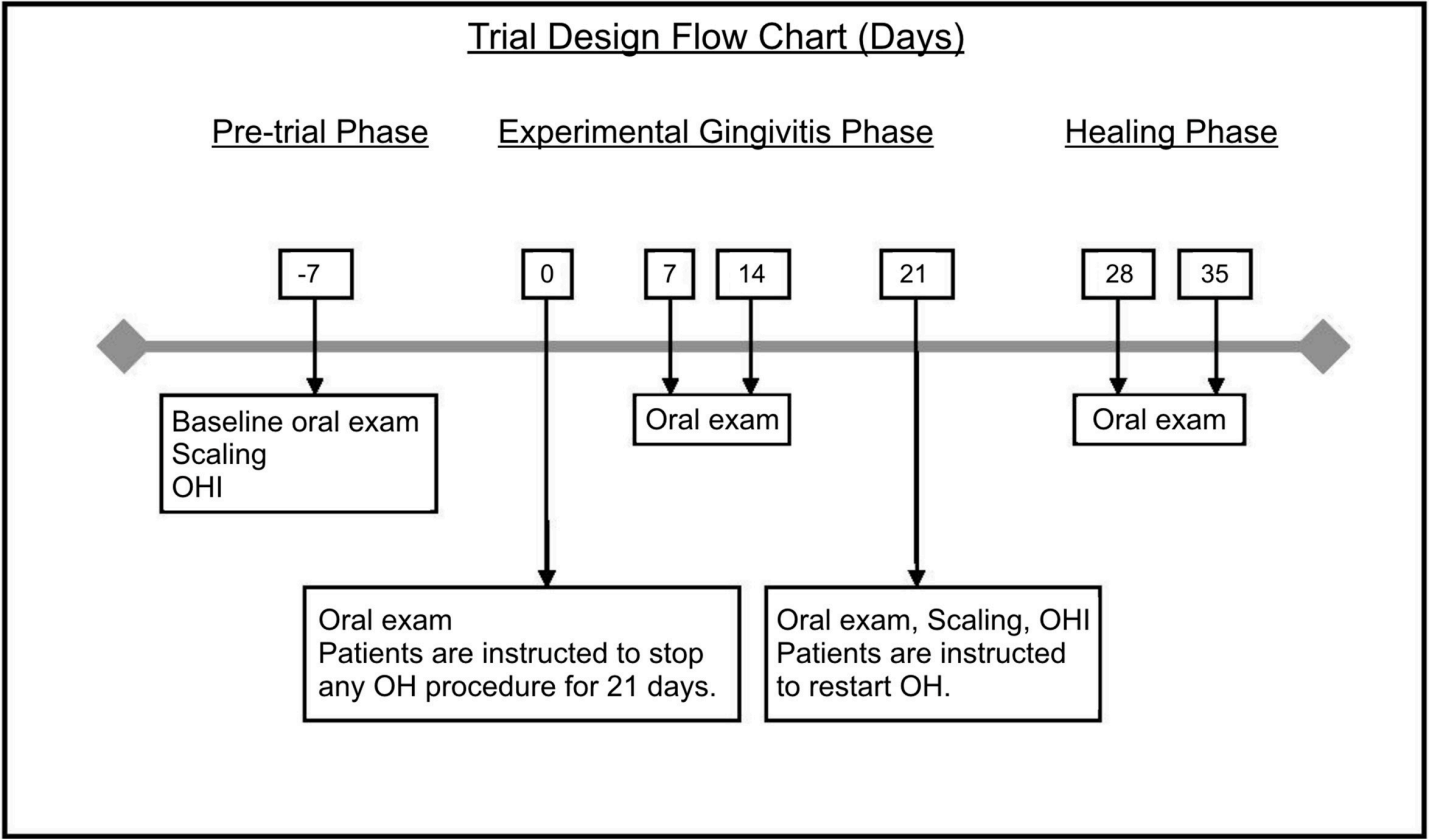
**Experimental gingivitis trial design flow chart (days)**. Stimulated whole saliva samples were collected at each time point.

Twenty-one day experimental gingivitis (EG) model was used to investigate protein dynamics at health (day 0 of EG) and GI (day 21 of EG). Study design and goals were presented to all participants, and consent was obtained in writing. The study was approved by the Scientific and Ethics Review Boards at the University of Toronto (protocols #24295/#24567) and conducted in the Graduate Periodontics Clinic at the Faculty of Dentistry from September–December 2012. Five periodontally healthy participants (2 males, 3 females, age range 20–36) completed EG trial. All study participants were systemically healthy and non-smokers. Participants completed baseline periodontal exam and professional scaling by a registered dental hygienist, followed by 7 days of enhanced oral hygiene (pre-study hygiene phase). On day 0 of EG, full periodontal exam was completed, and participants were asked to refrain from any oral hygiene procedures (including brushing, flossing, use of mouthwash, and gum chewing) for the remainder of the trial. EG inflammatory phase was concluded after 21 days. Oral neutrophils and WS samples were collected and clinical parameters measured on days 0, 7, 14, 21, 28, and 35. Participants received scaling and oral hygiene instructions. Participants were followed for 2 weeks during healing phase (Figure 1). During the trial, participants were followed weekly. Full periodontal exam and sample collection was completed at each visit. EG participants were used both as healthy control group (EG0–day 0 samples) and GI group (EG21–day 21 samples).

### Oral neutrophil quantification

Oral neutrophil quantification was completed utilizing hemocytometer technique, as previously described (Landzberg et al., 2015). Rinse samples for oral neutrophil cell counts were collected prior to any periodontal instrumentation to avoid sample contamination with blood. Subjects were asked to rinse with 10 mL of 0.9% isotonic sodium chloride solution (Baxter, Toronto, ON) for 30 s, and then expectorate into a 50 mL polypropylene tube (Sigma-Aldrich, St. Louis, MO). A 500 μl sample was separated into an 1.5 ml polypropylene tube and fixed with 50 μl with 37% formaldehyde (Sigma-Aldrich, St. Louis, MO). Samples were kept in 4°C until analysis. All cell counts were completed by the same examiner (GMA). Samples were centrifuged at 1139 × g for 5 min (Hettich Zentrifugen, Tuttlingen, Germany). The supernatant was removed and the pellet was resuspended in 100 μL of phosphate buffered saline (PBS. Sigma-Aldrich, St. Louis, MO). One microliter acridine orange (Sigma Chemical, Burlington, ON, Canada) was added to the cell suspension. Acridine orange is a fluorescent nucleic acid marker. Its interaction with DNA and RNA allows the identification of neutrophils under fluorescence microscope. Following acridine orange staining, samples were incubated, light protected, for 15 min at room temperature. A 10 μL aliquot of this suspension was loaded on to a hemocytometer (Bright-Line; Hausser Scientific, Horsham, PA, USA), and the neutrophils were visually counted using fluorescence microscopy (Leitz Orthoplan Microscope; Leitz, Wetzlar, Germany). Neutrophils were counted and quantified based on the standard protocol for hemocytometer use.

### Whole saliva (WS) sample collection and preparation

Stimulated WS samples were collected prior to any periodontal instrumentation to avoid sample contamination with blood. Chewing has been demonstrated to increase GCF flow, therefore subjects were asked to chew on a 5 × 5 cm parafilm (around 1.4 g, Parafilm M, Brand, Wertheim, Germany). This allowed for an increased GCF component in the analyzed WS samples (Griffiths, 2003). All EG sample collection took place in the morning, at the same time for each subject. Stimulated saliva in the first 30 s was discarded (swallowed). Patients were then asked to expectorate stimulated saliva into a 50 ml polypropylene tube (Sigma-Aldrich, St. Louis, MO) until 15 ml were collected. Salivary flow rate (ml/min) was acquired for each saliva collection. Saliva samples were placed on ice until aliquoting, which was completed within 3 h of sample collection. Samples were aliquoted into 1 ml portions. Three 1 ml samples were kept at −80°C until analysis and analyzed as whole saliva samples. Six 1 ml samples were centrifuged at 14,000 g over 20 min (Eppendorf Centrifuge 5415R, Eppendorf, Hauppauge, NY). Supernatant was separated from pellet, and both were kept at −80°C until analysis. The remaining 6 ml were stored at −80°C and were used as a reservoir for additional testing (Siqueira et al., 2004). Our goal was to establish a disease-specific proteome profile and a trend that characterizes the inflammatory and healing phases of gingival inflammation rather than individual investigations. Therefore, samples for each of the study groups were pooled together (Siqueira et al., 2012). As previously described (Silva-Boghossian et al., 2013), pooled samples were denatured and reduced for 2 h by buffer containing 4M urea, 10 mM dithiothreitol (DTT), and 50 mM ammonium bicarbonate (NH_4_HCO_3_), pH 7.8. Following dilution with 50 mM ammonium bicarbonate and the addition of 2% w/w sequencing-grade trypsin (Promega, Madison, WI), tryptic digestion was carried out for 18 h at 37°C.

### Liquid chromatography electrospray ionization tandem mass spectrometry (LC-ESI-MS/MS)

Mass spectrometric analyses were carried out with a LTQ-Velos (Thermo Scientific, San Jose, CA, USA) which allows in-line liquid chromatography with the capillary fused silica C18 column (column length 10 mm, column ID 75 μm, 3 μm spherical beads and 100 Å pores size) linked to mass spectrometer using an electrospray ionization in a survey scan in the range of m/z values 390–2000 tandem MS/MS. All samples were dried by rotary evaporator and re-suspended in 20 μL of 97.5% H_2_O/2.4% acetonitrile/0.1% formic acid and then subjected to reversed-phase LC-ESI-MS/MS. The nano-flow reversed-phase HPLC was developed with linear 80-min gradient ranging from 5 to 55% of solvent B (97.5% acetonitrile, 0.1% formic acid) at a flow rate of 300 nL/min with a maximum pressure of 280 bar. Electrospray voltage and the temperature of the ion transfer capillary were 1.8 kV and 250°C, respectively. Each survey scan (MS) was followed by automated sequential selection of seven peptides for CID, with dynamic exclusion of the previously selected ions. The obtained MS/MS spectra were searched against human protein databases (Swiss Prot and TrEMBL, Swiss Institute of Bioinformatics, Geneva, Switzerland, http://ca.expasy.org/sprot/) using SEQUEST algorithm in Proteome Discoverer 1.3 software (Thermo Scientific, San Jose, CA, USA). Search results were filtered for a false discovery rate of 1% employing a decoy search strategy utilizing a reverse database. An additional inclusion criterion for positive identification of proteins was the same protein passing the filter score at least in two different MS analyses from a total of three MS analyses per condition.

### Relative proteome quantitation

For quantitative proteome analysis, three MS raw files from each pooled group were analyzed using SIEVE technology (Version 2.0 Thermo Scientific, San Jose, CA, USA) as previously described (Siqueira et al., 2012). Relative Proteome quantitation for day 0 vs. day 21 of EG, were carried out. Baseline samples were compared to each of the study groups. Initial alignment step was carried out using a single MS raw file belonging to the baseline group. This file was selected as the reference file and all of other files were adjusted accordingly. Following the alignment, the feature detection and integration (or framing) process was performed through the “Frames from MS2 Scans” feature, using the MS level data. This framing process employs only MS mass-to-charge ratio (m/z) values that were associated with MS2 scan only. The parameters used consisted of a frame m/z width of 1500 ppm and a retention time width of 1.75 min. Peak integration was performed for each frame and these values were used for statistic analysis. Next, peptide sequences obtained from the database search using SEQUEST algorithm were imported into SIEVE. Peptides were grouped into proteins and a protein ratio and *p*-value were calculated. SIEVE uses a weighted average of the peptide intensities for the protein calculation. By using the weighted average, peptides with lower variance in their intensity measurements have a higher weight on the overall protein ratio. This is done to decrease variance in protein level quantities based on variance of the peptides that compose the proteins (Siqueira et al., 2012).

### Correlation assessment between salivary proteins abundance and oral neutrophils quantification

Pearson correlation was calculated for each of the whole saliva cytoprotective proteins and the oral neutrophil using JMP software (SAS, Cary, NC). The association between oral neutrophil levels and the level of cytoprotective proteins was tested through correlation analysis during the experimental gingivitis phase (days 0–21), and the healing period which followed (days 28–35).

## Results

**Figure 2:**
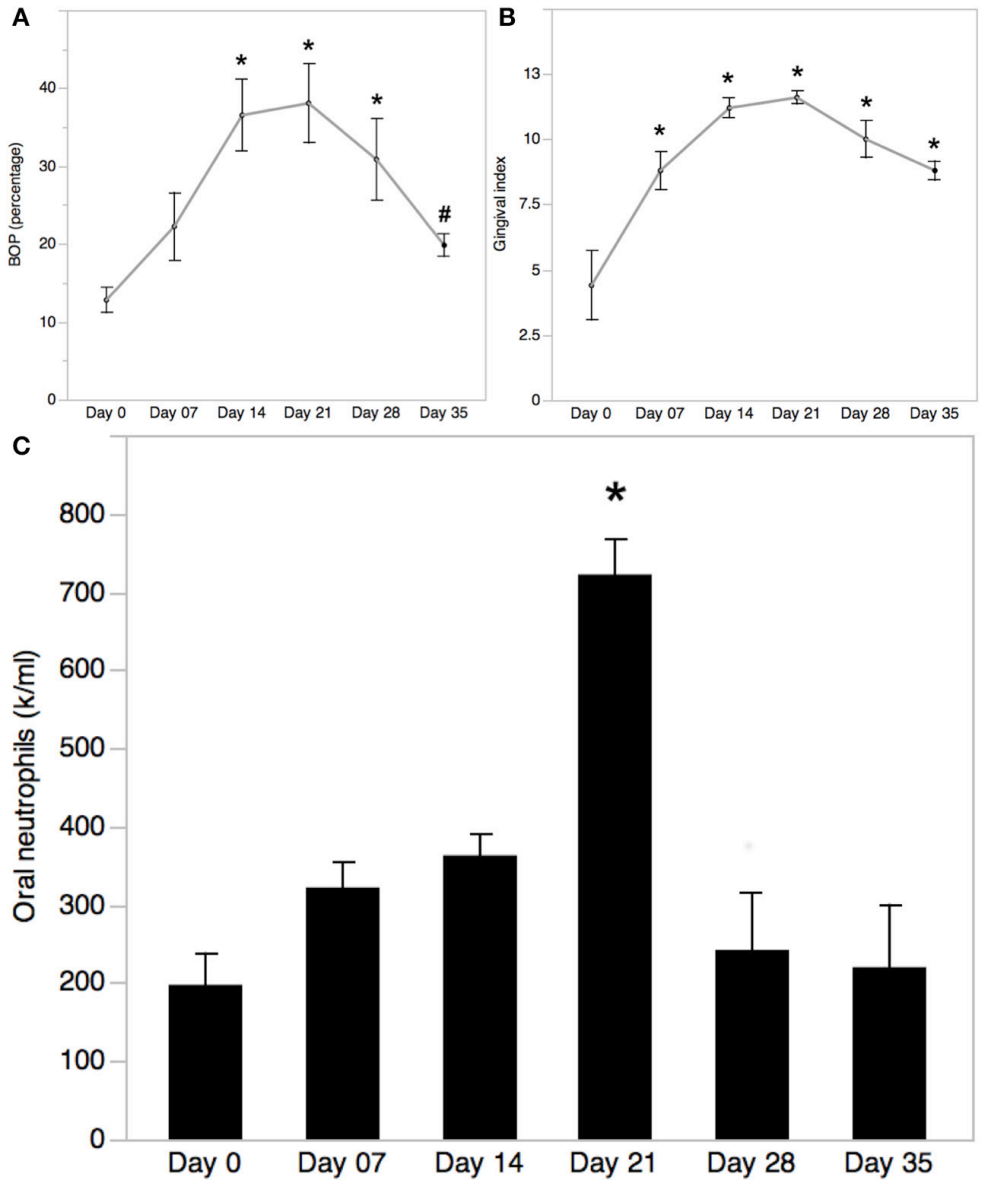
**Bleeding on Probing (BOP) trend in experimental gingivitis**. **(A)** Bleeding on probing (BOP) was measured at baseline (day 0) and on days 7, 14, 21, 28, and 35 during EG. Mean ± SEM; *n* = 5; One-way ANOVA with Turkey-Kramer HSD, ^*^p < 0.05 vs. day 0, ^#^*p* < 0.05 vs. day 21. **(B)** Gingival index was measured at baseline (day 0) and on days 7, 14, 21, 28, and 35 during EG. Mean ± SEM; *n* = 5; One-way ANOVA with Turkey-Kramer HSD, ^*^p < 0.05. **(C)** Oral neutrophils isolated from oral rinses collected on days 0, 7, 14, 21, 28, and 35 of EG were quantified using a hemocytometer. Cell numbers were normalized to the rinse volume collected. Mean ± SEM; *n* = 5; One-way ANOVA with Turkey-Kramer HSD; ^*^p < 0.05.

Worsening of clinical inflammatory parameters and biofilm accumulation were evident in all EG participants by day 21, including Bleeding on Probing (BOP), and Gingival Index (Löe, 1967). Changes in clinical parameters were detected by day 7, and increased significantly by day 14 reaching a peak on day 21 and returned to baseline levels by day 35, after subjects returning to regular OH on day 21. BOP was significantly higher on days 14 and 21 compared to day 0 and 35 (day 0, 12.8 ± 1.6 %; day 7, 22.2 ± 4.4 %; day 14, 36.5 ± 4.6 %; day 21, 38.1 ± 5.1 %; day 28, 30.9 ± 5.2 %; day 35, 19.8 ± 1.4 %; Figure 2A). Similarly, GI increased progressively during the inflammatory phase and decreased in the healing phase (day 0, 4.4 ± 1.3; day 7, 8.8 ± 0.7; day 14, 11.2 ± 0.4; day 21, 11.6 ± 0.24; day 28, 10 ± 0.7; day 35, 8.8 ± 0.4; Figure 2B). A significant increase in oral neutrophil numbers was found on day 21 of the inflammatory phase of GI compared to baseline (Figure 2C). Oral neutrophil counts decreased in the resolution phase by day 35, reaching levels close to baseline (day 0, 198 ± 40 × 10^4^; day 7, 322 ± 32 × 10^4^; day 14, 363 ± 27 × 10^4^; day 21, 722 ± 44 × 10^4^; day 28, 242 ± 73 × 10^4^; day 35, 220 ± 79 × 10^4^).

Salivary flow rate (ml/min) measured during all time-points of the study demonstrated no statistical significance difference among the groups (day 7, 1.23 ± 0.08 day 1.23 ± 0.21; day 7, 1.21 ± 0.07; day 14, 1.11 ± 0.09; day 21, 1.11 ± 0.18; day 28, 1.16 ± 0.19; day 35, 1.21 ±0.24).

Proteome data analysis revealed that 89 proteins showed significant level changes (at *p* < 0.05; Table 1) during EG. Twenty-one percent of these proteins (25 proteins) demonstrated a significant increase of at least 2 fold by day 21 compared to baseline (Table 2). Serum albumin levels identified in WS samples significantly increased during the inflammatory EG phase (2.3 fold increase). Furthermore, collagen fragment abundance increased significantly (7.35 fold increase), in line with the histologic findings previously described (Page and Schroeder, 1976).

**Table 1:** **Proteins with significant change in WS levels during the inflammatory phase of EG (Day 0 vs. Day 21)**.

**Accession**	**Protein name**	**Fold change**	***p*-Value**
A7E2D6	NAV2 protein	1.52	0.000
A8K6R0	cDNA FLJ75726, highly similar to Homo sapiens basic leucine zipper nuclear factor 1 (JEM-1) (BLZF1), mRNA	1.92	0.004
B0I1T1	MYO1F variant protein	1.71	0.013
B0ZBF6	Mineralocorticoid receptor	1.07	0.007
B1AN48	Small proline-rich protein 3	1.03	0.000
B3KTH9	cDNA FLJ38275 fis, highly similar to GAS2-like protein 3	2.31	0.004
B3KVS4	cDNA FLJ41312 fis, clone BRAMY2042804, highly similar to Homo sapiens zinc finger RNA binding protein (ZFR), mRNA	1.81	0.000
B3KXQ8	cDNA FLJ45892 fis, clone OCBBF3023175, highly similar to Protein neurobeachin	1.85	0.005
B4DG67	cDNA FLJ58842, highly similar to Homo sapiens zinc and ring finger 1 (ZNRF1), mRNA	1.72	0.008
B4DH81	cDNA FLJ61250, highly similar to Homo sapiens GTPase activating Rap/RanGAP domain-like 3 (GARNL3), mRNA	1.59	0.006
B4DSR5	Kinesin-like protein KIF3B	2.23	0.013
B4DSW4	cDNA FLJ51541, moderately similar to Transcription factor Sp8	1.64	0.048
B4DT16	B-cell lymphoma/leukemia 11A	1.71	0.000
B4DVQ0	cDNA FLJ58286, highly similar to Actin, cytoplasmic 2	1.75	0.005
B4DYH2	cDNA FLJ53243	1.72	0.012
B4DYQ3	cDNA FLJ60974, highly similar to Mediator of RNA polymerase II transcription subunit 12	1.69	0.009
B7ZAX4	cDNA, FLJ79338, highly similar to Krueppel-like factor 11	1.20	0.000
B7ZMD7	Amylase, alpha 1A	0.79	0.001
C3PTT6	Pancreatic adenocarcinoma unregulated factor	2.25	0.012
C8C504	Beta-globin	1.77	0.018
C9J185	Eukaryotic translation initiation factor 2-alpha kinase 3 (Fragment)	1.64	0.006
C9JAJ5	Putative uncharacterized protein LOC349136	2.18	0.036
C9JC68	Fetuin-B (Fragment)	1.09	0.009
D3DPB9	Nitric oxide synthase trafficker, isoform CRA_a	1.09	0.031
D3DRN4	KIAA1539, isoform CRA_a	4.72	0.003
D6RGV6	Serine/threonine-protein kinase Nek11	0.72	0.005
E5KRP6	Spastin	1.88	0.036
E7EMQ1	Carbonic anhydrase 6	2.23	0.039
E7ETI5	G-protein-coupled receptor 98	3.41	0.018
E9PAV3	Nascent polypeptide-associated complex subunit alpha	1.50	0.010
E9PIJ5	Transmembrane protease serine 13	2.21	0.000
F2Z2U9	Myosin-14	1.89	0.009
F4MH44	Ubiquitously transcribed tetratricopeptide repeat protein Y-linked transcript variant 22	1.68	0.010
F5H2N0	Zinc finger protein 574	1.37	0.028
F8W696	Apolipoprotein A-I	2.32	0.027
G3CIG0	MUC19 variant 12	1.21	0.000
H0Y3D5	Fibroblast growth factor receptor 4	1.72	0.022
H0YBS7	Gamma-aminobutyric acid receptor subunit alpha-6 (Fragment)	1.72	0.002
H0YD40	Collagen alpha-1 (XXVII) chain (Fragment)	7.35	0.001
H0YL38	Zinc finger protein 280D	1.82	0.032
H6VRF8	Keratin 1	1.00	0.001
O14513	Nck-associated protein 5	1.72	0.027
O15047	Histone-lysine N-methyltransferase SETD1A	1.61	0.012
O75443	Alpha-tectorin	1.62	0.009
O75592	Probable E3 ubiquitin-protein ligase MYCBP2	1.95	0.000
O95447	Lebercilin-like protein	1.66	0.001
O95613	Pericentrin	1.55	0.026
P01036	Cystatin-S	2.14	0.006
P01037	Cystatin-SN	2.33	0.046
P01833	Polymeric immunoglobulin receptor	1.10	0.007
P01834	Ig kappa chain C region	2.87	0.005
P01857	Ig gamma-1 chain C region	0.62	0.001
P01876	Ig alpha-1 chain C region	1.56	0.039
P02768	Albumin	2.33	0.022
P02774	Vitamin D-binding protein	2.78	0.001
P02788	Lactotransferrin	3.50	0.002
P02808	Statherin	1.23	0.001
P02814	Submaxillary gland androgen-regulated protein 3B	1.59	0.006
P04083	Annexin A1	4.15	0.002
P06733	Alpha-enolase	1.88	0.001
P10599	Thioredoxin	2.29	0.000
P15515	Histatin-1	1.29	0.000
P19961	Alpha-amylase 2B	0.87	0.003
P20930	Filaggrin	1.87	0.017
P22748	Carbonic anhydrase 4	0.64	0.002
P27482	Calmodulin-like protein 3	2.11	0.000
P61626	Lysozyme C	1.67	0.003
Q05BP9	OLIG2 protein	2.47	0.042
Q12912	Lymphoid-restricted membrane protein	1.21	0.001
Q12955	Ankyrin-3	0.07	0.001
Q14118	Dystroglycan	0.68	0.005
Q14484	Beta-globin	3.75	0.001
Q14515	SPARC-like protein 1	2.10	0.005
Q14C71	GLRA1 protein	1.41	0.032
Q16378	Proline-rich protein 4	0.93	0.008
Q502W4	IGKC protein	1.57	0.013
Q569J1	IGHA1 protein	1.33	0.026
Q59FG1	Calcium channel, voltage-dependent, alpha 1E subunit variant (Fragment)	1.49	0.012
Q5H9S0	Putative uncharacterized protein DKFZp781N1974	1.57	0.000
Q5JPC9	ABI gene family, member 3 (NESH) binding protein, isoform CRA_d	1.82	0.009
Q6N092	Putative uncharacterized protein DKFZp686K18196 (Fragment)	1.27	0.008
Q6PL43	Uncharacterized protein	1.96	0.014
Q6W4X9	Mucin-6	2.13	0.045
Q86UD4	Zinc finger protein 329	1.60	0.002
Q8TDL5	Long palate, lung and nasal epithelium carcinoma-associated protein 1	0.38	0.001
Q8WXI7	Mucin-16	1.99	0.000
Q96AY4	Tetratricopeptide repeat protein 28	2.20	0.020
Q96FS4	Signal-induced proliferation-associated protein 1	1.40	0.030
Q9BZG5	Androgen receptor	2.21	0.002

**Table 2:** **Proteins with significant increase of >2 fold during the inflammatory phase of EG**.

**Accession**	**Protein name**	**Fold change**	***p*-Value**
B3KTH9	cDNA FLJ38275 fis, highly similar to GAS2-like protein 3	2.31	0.004
B4DSR5	Kinesin-like protein KIF3B	2.23	0.013
C3PTT6	Pancreatic adenocarcinoma unregulated factor	2.25	0.012
C9JAJ5	Putative uncharacterized protein LOC349136	2.18	0.036
D3DRN4	KIAA1539, isoform CRA_a	4.72	0.003
E7EMQ1	Carbonic anhydrase 6	2.23	0.039
E7ETI5	G-protein-coupled receptor 98	3.41	0.018
E9PIJ5	Transmembrane protease serine 13	2.21	0.000
F8W696	Apolipoprotein A-I	2.32	0.027
H0YD40	Collagen alpha-1 (XXVII) chain (Fragment)	7.35	0.001
P01036	Cystatin-S	2.14	0.006
P01037	Cystatin-SN	2.33	0.046
P01834	Ig kappa chain C region	2.87	0.005
P02768	Albumin	2.33	0.022
P02774	Vitamin D-binding protein	2.78	0.001
P02788	Lactotransferrin	3.50	0.002
P04083	Annexin A1	4.15	0.002
P10599	Thioredoxin	2.29	0.000
P27482	Calmodulin-like protein 3	2.11	0.000
Q05BP9	OLIG2 protein	2.47	0.042
Q14484	Beta-globin	3.75	0.001
Q14515	SPARC-like protein 1	2.10	0.005
Q6W4X9	Mucin-6	2.13	0.045
Q96AY4	Tetratricopeptide repeat protein 28	2.20	0.020
Q9BZG5	Androgen receptor	2.21	0.002

*Highlighted proteins are proteins considered cytoprotective proteins*.

**Figure 3:**
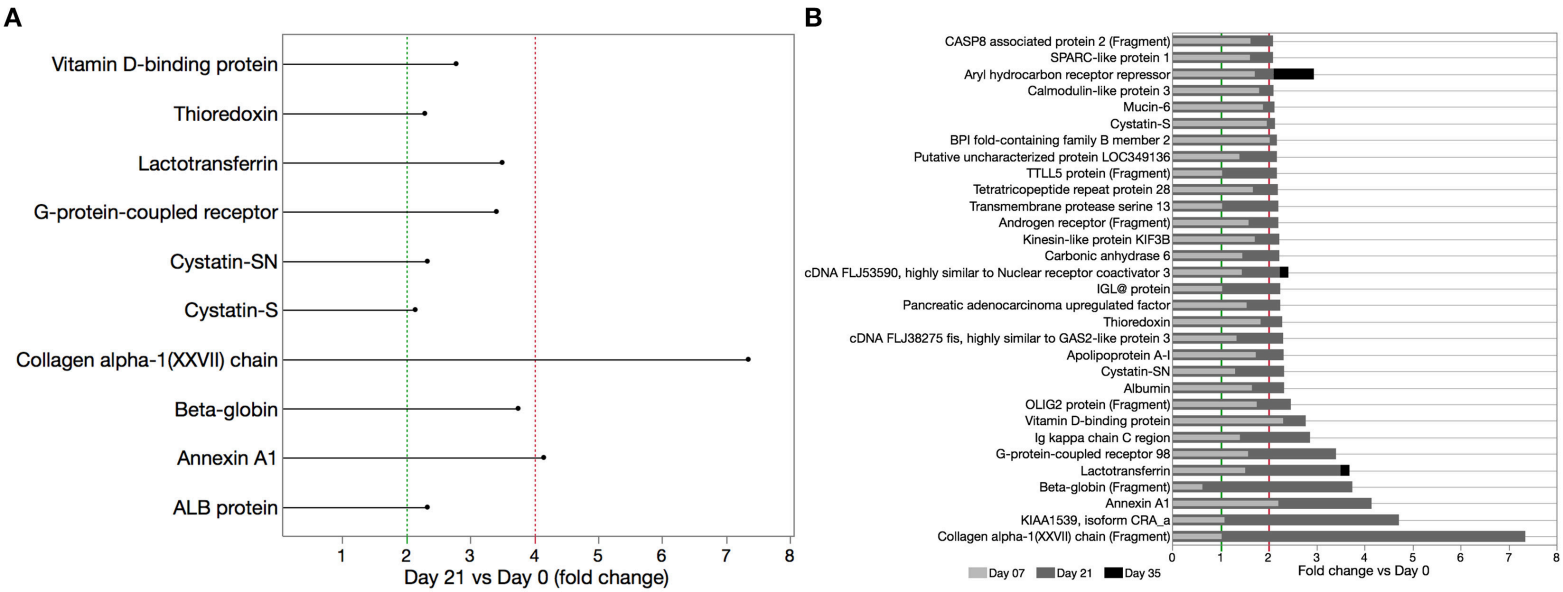
**Cytoprotective proteins significantly increased during EG (day 21 vs. day 0)**. **(A)** WS samples were analyzed by LC-ESI-MS/MS and relative proteome analysis performed based on peptide sequences obtained from the database search using SEQUEST algorithm after peak integration from acquired frames and SIEVE-assisted recognition of proteins form peptide sequences. **(B)** Time course of salivary protein levels for select proteins that increased by >2 fold on day 21 vs. baseline. With exception of aryl hydrocarbon receptor repressor, cDNA FLJ3590 fis and lactotransferrin all proteins with >2 fold increase in the inflammatory phase were reduced by day 35, 2 weeks after oral hygiene was resumed.

Significant increases in several cytoprotective proteins were observed during the inflammatory phase, including proteins involved in inflammatory regulation (Annexin A1 and Vitamin D binding protein–4.15 and 2.78 fold increase, respectively), antibacterial (Lactotransferrin–3.5 fold increase), antioxidants (β-globin and Thioredoxin–3.75 and 2.29 fold increase, respectively), and protease inhibitor proteins (Cystatin SN, Cystatin S–2.33 and 2.14 fold increase, respectively; Figure 3A). Abundance of these cytoprotective proteins went back to baseline levels in the resolution phase of EG. Eighteen of the 25 salivary proteins that increased by >2 fold by day 21, followed a trend of reduction in the resolution phase. Aryl hydrocarbon receptor repressor, cDNA FLJ3590 fis and lactotransferrin increased further by day 35 (Figure 3B).

**Figure 4:**
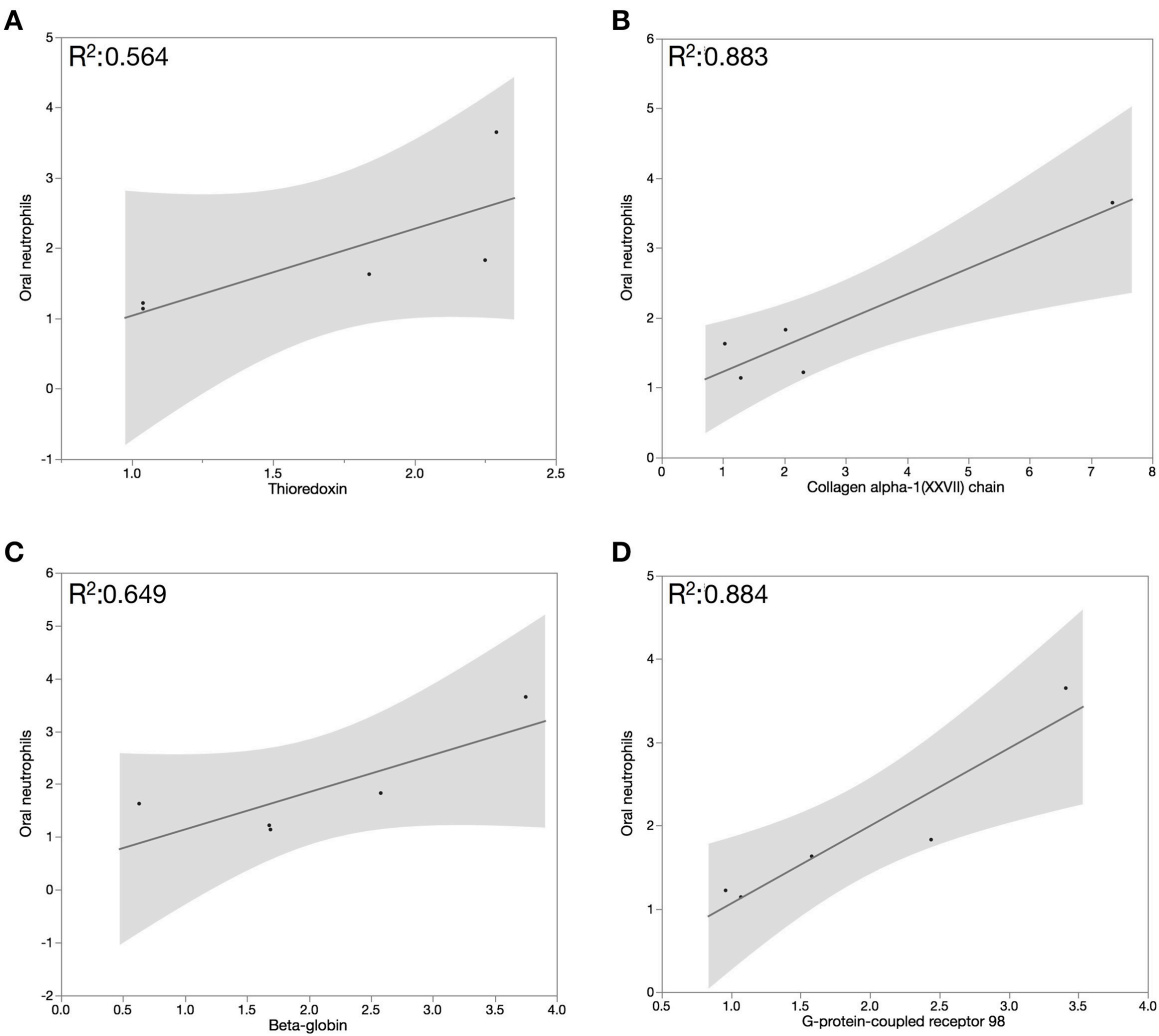
**Pearson correlation assessment between salivary proteins abundance and oral neutrophils quantification demonstrated that oral neutrophil numbers correlated moderately with salivary β-globin (A) and thioredoxin (B) and strongly with (C) collagen alpha-1(XXVII) chain and (D) G-protein coupled receptor 98 in the inflammatory and resolution phases of GI**.

The inflammatory response observed during gingivitis is mediated by neutrophils migrating from the blood stream to the gingival tissues and in to the oral cavity through the GCF. We speculate that the increase in oral neutrophil levels during the experimental gingivitis phase is responsible in part for the observed increase in whole saliva cytoprotective proteins. Pearson correlation assessment between salivary proteins abundance and oral neutrophils quantification demonstrated that oral neutrophil numbers correlated moderately with salivary β-globin and thioredoxin (*R*^2^ = 0.649 and 0.564, respectively), and strongly with collagen alpha-1(XXVII) chain and G-protein coupled receptor 98 (*R*^2^ = 0.883 and 0.884, respectively), in the inflammatory and resolution phases of GI (Figure 4).

## Discussion

The comparison of proteome profile at baseline and day 21 of EG demonstrated that several salivary proteins increased by at least 2 fold during the inflammatory phase of GI. Among these proteins, we identified 10 cytoprotective proteins. The overall increase in these proteins, which are mostly involved in tissue protection and inflammatory control, suggests that activation of protective pathways, including antibacterial activity, regulation of the inflammatory process, antioxidants, and protease inhibitors characterizes host responses of healthy individuals to increasing bacterial burden on the gingiva. The moderate correlation between oral neutrophil numbers and salivary β-globin and thioredoxin in the inflammation and resolution phases of GI indicates that salivary antioxidants may not be the primary source of protection against neutrophil-mediated oxidative damage in periodontal tissue inflammation. The inflammatory response observed during gingivitis is mediated by neutrophils migrating from the blood stream to the gingival tissues and in to the oral cavity through the GCF. We speculate that the increase in oral neutrophil levels during the experimental gingivitis phase is responsible in part for the observed increase in whole saliva cytoprotective proteins. The association between oral neutrophil levels and levels of proteins of interest was tested through correlation analysis during the experimental gingivitis phase (days 0–21), and the healing period which followed (days 28–35). In general, we found moderate to strong correlations between oral neutrophil numbers and levels of several salivary cytoprotective proteins in inflammation and resolution of gingivitis.

Lactotransferrin (also known as lactoferrin) is an iron-binding protein (Brock, 2002; Ammons and Copié, 2013), found in serum and secreted fluids, and also present in specific neutrophil granules (Masson et al., 1969). Its antibacterial properties result from bacteriostatic activity, directly related to its iron-chelating capacity; and bactericidal activity, as a result of a direct interaction between the protein and bacteria where the N-terminal region of LTF, known as lactoferricin, can disrupt or possibly even penetrate bacterial cell membranes (Brock, 2002). Anti-biofilm activity of LTF was also demonstrated, as it inhibits biofilm formation and reduces the established biofilm of oral bacteria at physiological concentrations (Wakabayashi et al., 2010; Ammons and Copié, 2013). It has been speculated that LTF inhibits biofilm formation and disrupts existing biofilm by preventing bacterial adhesion or stimulating bacterial motility. Similar to our current findings, significant increase in LTF abundance was previously demonstrated in GCF samples during EG (15). The reported antibacterial and anti-biofilm properties of LTF can explain its increased abundance during EG, as an innate protective response to bacterial accumulation. Due to these protective properties, LTF was suggested as a potential treatment for CP (Wakabayashi et al., 2010).

In addition to biofilm control-associated factors, several anti-inflammatory proteins were changed during the course of inflammation-resolution in our EG study. Two inflammation regulating proteins were increased in the inflammatory phase of EG: Annexin A1 and Vitamin D binding protein. Annexins are a family of calcium and phospholipid binding proteins (Gerke and Moss, 2002), which dampen the inflammatory response via inhibition of neutrophil activation (Chatterjee et al., 2005). Annexin A1 levels in GCF were previously demonstrated to be stable during 21 days EG, opposing our current findings (Grant et al., 2010). One possible explanation is that we used WS samples and not GCF samples. Therefore, it is possible that salivary sources contribute to annexin A1 production and its involvement in regulating gingival inflammation.

Vitamin D binding protein (DBP) is a multifunctional protein found in plasma. Its ability to bind to vitamin D (calcitriol) and its metabolites and to transfer them to target cells has a major role in the involvement of vitamin D in inflammatory regulation. Vitamin D was shown to be involved in both the innate and adaptive immune systems, with vitamin D insufficiency being linked to many inflammatory disorders, including periodontal diseases. It has been suggested that vitamin D may act similarly to cytokines, and regulate the inflammatory process by several mechanisms: stimulating phagocytosis and antibody-presenting actions to enhance the initial immune response. As the inflammatory process progresses, vitamin D plays a role in inhibition of T-cell proliferation and thus inflammatory resolution (Stein et al., 2014). DBP itself was demonstrated to have significant neutrophil chemotactic activity *in vivo* where DBP knock-out mice demonstrated significant decreases in neutrophil recruitment to the site of infection when compared to the wild type group. Exogenous addition of DBP was shown to restore neutrophil response (Trujillo et al., 2013). Our observation of increased DBP levels during EG may suggest an increased abundance of vitamin D during this phase. High vitamin D levels were suggested to reduce inflammation during gingivitis (Dietrich et al., 2005).

Increases in oxidative stress, where reactive oxygen species (ROS) levels exceed antioxidant levels, have been shown to directly contribute to periodontal inflammation and connective tissue breakdown during periodontal disease. The antioxidant defense systems have an important role in balancing physiological oxidative stress (Chapple and Matthews, 2007). Total antioxidant capacity (TAC) levels were found to be significantly lower in periodontitis patients when compared to healthy controls (Chapple et al., 2007). Our current findings demonstrate an increase in antioxidant proteins–β-globin and Thioredoxin during the development of EG, suggesting an additional protective mechanism during this reversible phase of periodontal disease.

The globin superfamilies are hemeproteins which can be found in all known life forms. Its common role in O2 transport in vertebrate erythrocytes is recognized as a relatively recent adaptation from its more primative functions in non-erythroid cells, including iron metabolism regulation, intracellular oxygen transport, oxygen sensing, NO scavenging, and hydrogen peroxide scavenging (Vinogradov and Moens, 2008). Several human studies demonstrated that hemoglobin overexpression reduces oxidative stress, suggesting its cytoprotective role as an antioxidant (Liu et al., 2011; Li et al., 2013). Increased β-globin levels were demonstrated in mice macrophages treated with lipopolysaccharide and interferon-γ (Liu et al., 1999). In-line with our current findings, salivary proteome analysis of samples collected from gingivitis patients with gingivitis revealed increase in both α- and β-globin compared to healthy controls (Gonçalves Lda et al., 2011).

Thioredoxin (Trx) is an intra-cellular protein that together with thioredoxin reductase and NADPH, comprises the thioredoxin system. This system has been shown to play a key role in many intra-cellular pathways including H2O2 as a means to reduce oxidative stress (Holmgren and Lu, 2010). The cytoprotective effects of Trx were demonstrated in transgenic mice overexpressing human Trx1 that are resistant to oxidative stress conditions, and are more resistant to inflammation (Yoshida et al., 2005). In humans, increased extracellular Trx levels were reported for various systemic conditions, including rheumatoid arthritis (RA; Maurice et al., 1999). Our current results demonstrate an increase in Trx during EG, supporting its protective role during this reversible inflammatory phase.

Several proteolytic enzymes have been demonstrated to play a key role in periodontal tissue destruction. The main sources for these enzymes are the neutrophils that populate the periodontal pocket and produce proteolytic enzymes as part of the non-oxidative killing function (Meyer-Hoffert and Wiedow, 2011). We identified a significant increase in the protease inhibitor proteins Cystatin SN and Cystatin S during EG. Cystatins SN and S were first identified in saliva, and have been identified in other secreted fluids. Their production in secretory glands suggests their role as cytoprotective inhibitors of exogenous cysteine peptidases (Abrahamson et al., 2003). Cystatin S levels in GCF have been demonstrated to be stable during the 21 days EG model, which once again is in contrast to our current findings (Grant et al., 2010). In a different study, GCF proteome analysis reported decrease in cystatin S abundance in GCF samples obtained from GI patients (Huynh et al., 2014). Salivary glands contribute to cystatin S production, which can explain the differences in our analysis of WS samples, as opposed to GCF samples. In support of our findings, cystatin activity was demonstrated to increase in WS samples obtained from GI patients (Henskens et al., 1993), as well as in inflamed gingival tissues (Babnik et al., 1988).

The increase in cytoprotective proteins during the development of EG, suggests that production of proteins that can dampen the effector proteins involved in the inflammatory process is important during inflammatory diseases including GI (Figures 3, 4). Similar findings were reported in a human tears study, which demonstrated increase in cystatin S and lactotransferrin levels in tears collected from patients with autoimmune conditions (Katunuma et al., 2003). Interestingly, reduced levels of several of these cytoprotective proteins were previously reported in patients diagnosed with CP. Reduced cystatins (S, SN, SA) levels were previously reported in WS samples obtained from CP patients when compared to healthy controls (Henskens et al., 1996; Ito et al., 2008; Gonçalves Lda et al., 2010). Reports of LTF levels during CP were inconsistent: WS proteome analysis demonstrated a 2-fold increase in LTF levels when compared to healthy controls (Salazar et al., 2013). Contrary to this report, low levels of LTF were demonstrated in WS samples obtained from patients diagnosed with *Aggregatibacter actinomycetemcomitans*-associated periodontitis (Groenink et al., 1999), and in WS samples obtained from patients diagnosed with generalized aggressive periodontitis (GAgP) when compared to healthy controls (Wu et al., 2009). The same study also showed increased vitamin D binding protein levels in GAgP patients when compared to healthy controls (Wu et al., 2009).

Several theories may explain the reduced levels of protective proteins during CP: as the inflammatory process progresses, increased levels of endogenous and exogenous ROS and proteases may lead to damage of the cytoprotective proteins. A second theory suggests that once bacterial and inflammatory overload reach a specific threshold, the cytoprotective mechanisms are reduced. This threshold can vary between subjects, and may be responsible for the observed variability in the development of CP among subjects. The findings of the present study demonstrate that in the initial inflammatory phase of GI levels of salivary cytoprotective proteins are increased and that they decrease as inflammation resolves.

## Conclusions

We were able to demonstrate in the current pilot study a significant increase in several cytoprotective proteins during the inflammatory phase of GI. Further, we found that levels of these proteins decreased in the healing phase of GI indicating a dose-response relationship with gingival inflammation-resolution. The increase in cytoprotective proteins in onset of GI may prevent periodontal tissue destruction and clinical attachment loss in patients at risk of developing CP due to other host and environmental factors. Future studies to investigate whether these proteins can serve as biomarkers for GI progression to CP are necessary.

## Author contributions

GA, CS, WS, MG contributed to conception or design. GA, CS, WS, MG, YX, EM, KC contributed to acquisition, analysis, or interpretation. GA, CS, WS, MG drafted the manuscript. GA, CS, WS, MG, YX, EM, KC critically revised the manuscript. GA, CS, WS, MG, YX, EM, KC gave final approval. GA, CS, WS, MG, YX, EM, KC agrees to be accountable for all aspects of work ensuring integrity and accuracy.

### Conflict of interest statement

The authors declare that the research was conducted in the absence of any commercial or financial relationships that could be construed as a potential conflict of interest.
